# Endophytic *Aspergillii* and *Penicillii* from medicinal plants: a focus on antimicrobial and multidrug resistant pathogens inhibitory activity

**DOI:** 10.5114/bta.2024.135644

**Published:** 2024-03-29

**Authors:** Jendri Mamangkey, Lucas William Mendes, Apon Zaenal Mustopa, Adrian Hartanto

**Affiliations:** 1 Department of Biology Education, Faculty of Education and Teacher Training, Universitas Kristen Indonesia, Jakarta, Indonesia; 2 Cell and Molecular Biology Laboratory, Center for Nuclear Energy in Agriculture (CENA), University of São Paulo, Piracicaba, São Paulo, Brazil; 3 Research Center for Genetic Engineering, Research Organization for Life Sciences and Environment, National Research and Innovation Agency (BRIN), KST Soekarno, Cibinong, Bogor, Indonesia; 4 Department of Biology, Faculty of Mathematics and Natural Sciences, Universitas Sumatera Utara, Medan, Indonesia

**Keywords:** endophytic fungi, antibiotics, bioactive compounds

## Abstract

The rise of multidrug resistance among microorganisms, where they develop resistance against formerly efficacious drugs, has led to increased disease prevalence and mortality rates, posing a growing challenge. Globally, antibiotic resistance has made a significant impact, causing millions of fatalities each year. Endophytic fungi have gained considerable attention in research due to their potential to produce a wide variety of secondary metabolites, including natural substances with antimicrobial capabilities. The genera *Aspergillus* and *Penicillium* stand out as the most prevalent species of endophytic fungi. Filamentous fungi, such as these are responsible for the production of 45% of known microbial metabolites. This review focuses on exploring the bioactive substances produced by endophytic fungi from these two genera, particularly in conjunction with medicinal plants. Emphasis is placed on their antimicrobial activity and their ability to inhibit multidrug-resistant pathogens. As the need for alternative treatments to combat drug-resistant infections continues to grow, endophytic fungi have the potential to provide a valuable source of bioactive molecules for medical applications.

## Introduction

Multidrug resistance (MDR) is a phenomenon in which microorganisms develop resistance to drugs, even if they were once sensitive to them. This resistance contributes to increased disease and mortality rates (Tanwar et al., 2014). The search for new medications to treat health issues and infections is crucial, particularly in light of the global issue of antibiotic resistance. The impact of antibiotic resistance has been significant, with an expected 5.2 million people in the Western Pacific Region projected to succumb to drug-resistant bacterial infections by the end of 2030 (WHO, 2023). According to Pasrija et al. (2022), the percentage of infections caused by resistant microorganisms is 18% in Southeast Asia, 12% in the Western Pacific region, and 6% in Africa. Additionally, it is estimated that by 2050, MDR infections will lead to approximately 10 million deaths annually (Tagliaferri et al., 2020).

Endophytic fungi, residing in plant tissues without causing visible disease symptoms, have garnered significant attention for their ability to produce various secondary metabolites (Manganyi and Ateba, 2020; Mamangkey et al., 2022a). The natural substances produced by fungal endophytes exhibit a range of biological characteristics, including antimicrobial capabilities (Joo et al., 2021; Mamangkey et al., 2022b). Endophytes have been isolated from all studied plant species, and it is recognized that, among the estimated 300 000 known plant species on Earth, each hosts at least one endophytic resident. However, only a small fraction of plant species (approximately 1–2%) have been investigated for their endophytic populations (Strobel, 2018). Endophytes are located in various parts of the plant host, including single cells along vascular tissues and clusters inside certain epidermal cells, with uneven distribution within host tissue (de Souza et al., 2004). The need for an alternative to chemosynthetic medications for treating human diseases has grown, highlighting the importance of bioactive molecules produced by endophytes to combat the advent of new drug-resistant infections (Kraupner et al., 2018; Bengtsson-Palme et al., 2018; Adeleke and Babalola, 2020).

Common endophytic fungal species, particularly those belonging to the genera *Aspergillus* and *Penicillium*, are recognized as primary producers of certain toxic substances known as mycotoxins (Peilu et al., 2018). Despite this, these fungi also serve as abundant sources of bioactive compounds with potential medicinal benefits, as highlighted by George et al. (2019). Therefore, this study explores the different bioactive substances derived from endophytic fungi within the *Aspergillii* and *Penicillii*, particularly in conjunction with medicinal plants. The emphasis is on their significance in microbiology, including their antimicrobial activity and their ability to inhibit multidrug-resistant pathogens.

### Bioactive compounds

An antimicrobial is a substance capable of killing or inhibiting the growth of microorganisms, such as bacteria and fungi, thereby preventing their proliferation. Many researchers are currently investigating various extracts of endophytic fungi as potential antimicrobial agents. Filamentous fungi, including *Aspergillus* and *Penicillium*, which contribute to nearly 99% of all known fungal metabolites, are recognized for producing approximately 45% of known microbial metabolites (Berdy, 2012). This positions them as a particularly promising source of bioactive compounds for antimicrobial purposes. Endophytic fungi are known to synthesize a wide variety of chemicals, categorized into groups such as aliphatic metabolites, alkaloids, flavonoids, glycosides, lactones, phenyl propanoids, quinones, steroids, terpenoids, and xanthones (Zhang et al., 2006).

*Aspergillus* stands out as one of the most widely distributed genera of endophytic fungi, capable of forming symbiotic relationships with various organisms, including plants. Endophytic *Aspergillus* species have been shown to synthesize diverse secondary metabolites, including butenolides, alkaloids, terpenoids, cytochalasins, phenalenones, terphenyls, xanthones, sterols, diphenyl ether, and anthraquinones. These compounds hold substantial significance for the pharmaceutical and commercial sectors (El-hawary et al., 2020), making *Aspergillus* a valuable reservoir of bioactive molecules for medical applications. *Aspergillus* members are also gaining recognition as prolific producers of bioactive compounds, particularly antimicrobial agents. According to Domingos et al. (2022), *Aspergillus* is considered to possess the highest bioactive potential among all ascomycete fungi in the natural world. Extensive research has been conducted on species within this genus, known for generating metabolites of considerable economic and medicinal importance.

**Fig. 1:**
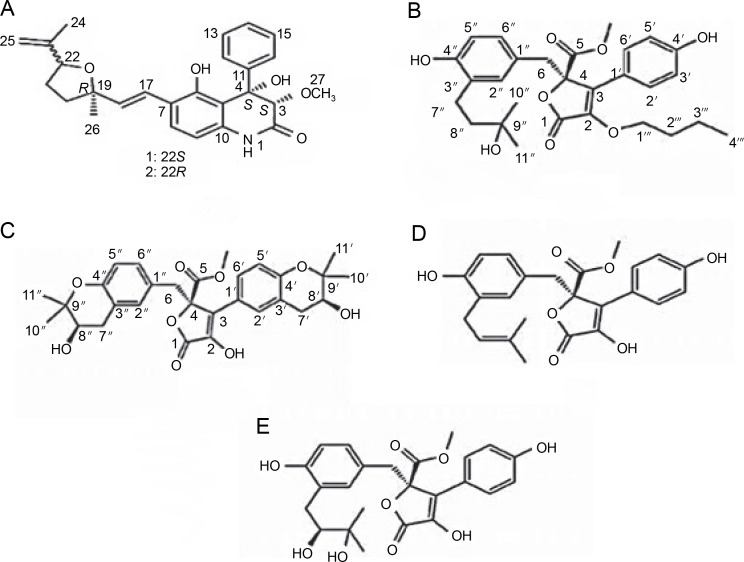
Structures of (A) aniduquinolone A (Ebada and Ebrahim, 2020), (B) aspernolides L, (C) aspernolides M, (D) butyrolactones I, and (E) butyrolactones VI (Ibrahim and Asfour, 2018)

The species *Aspergillus versicolor* has been reported to produce aniduquinolone A (Fig. 1A), an antibacterial compound inhibiting the growth of *A. versicolor* (Ebada and Ebrahim, 2020). Aniduquinolone A compound is extracted from ethyl acetate isolated from *A. versicolor* culture. Additionally, Ibrahim and Asfour (2018) demonstrated that *A. versicolor* produces aspernolides L, aspernolides M, butyrolactones I, and butyrolactones VI compounds (Fig. 1B–1E), all of which inhibit the growth of methicillin-resistant pathogenic bacteria, including *Candida albicans*, *Staphylococcus aureus*, *Pseudomonas aeruginosa*, and *Escherichia coli*.

The compound profile of *Aspergillus flavus* fermentation extract obtained using gas chromatography-mass spectrometry was reported to have various bioactive components. In this case, the main compounds identified were 4-nitrobenzoic acid, 3-chlorophenyl ester (27.23%), and (+)-salsolidine (21.82%) which are nitrobenzoate and alkaloid classes compounds, respectively. These compounds are known to have antimicrobial activities toward *Bacillus subtilis*, *E. coli* BL21, *Lactococcus lactis*, *S. aureus*, *Staphylococcus carnosus*, and *Staphlococcus simulans* (Chowdhury et al., 2018). In addition, Khattak et al. (2021) observed that *A. flavus* can produce (2E)-3-[(3S, 4R)-8-hydroxy-3, 4-dimethyl-1-oxo-3, 4-dihydro-1H-2-benzo-pyran-7-yl] prop-2-enoic acid compound with the formula of C_14_H_14_O_5_. This compound showed inhibition activity of 58.8% towards MDR strains of *S. aureus* and 28% towards *P. vulgaris*.

**Fig. 2:**
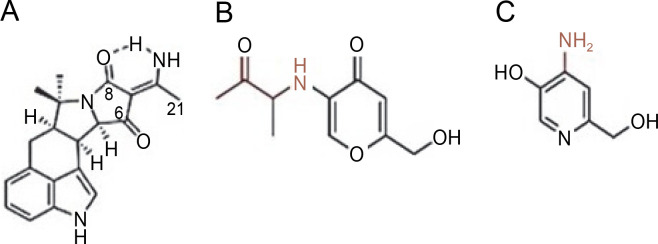
Structures of (A) 19-amino-19-dehydroxy 5-epi-α-cyclopiazonic acid, (B) 2-hydroxymethyl-5-(3-oxobutan-2-yl)aminopyran-4(4H)-one and (C) 4-amino-2 hydroxymethylpyridin-5-ol (He et al., 2021)

Endophytic *A. flavus* GZWMJZ-288, living in symbiosis with *Garcinia multiflora*, has been reported to produce valuable compounds, including 19-amino-19-dehydroxy 5-epiα-cyclopiazonic acid, 2-hydroxymethyl-5-(3-oxobutan-2-yl)-aminopyran-4(4H)-one, and 4-amino-2 hydroxymethylpyridin-5-ol (Fig. 2). All these compounds exhibit antimicrobial activity against pathogenic bacteria, namely *P. aeruginosa* ATCC10145, *E. coli* ATCC11775, *S. aureus* ATCC6538, *S. aureus* ATCC25923, and methicillin-resistant *S. aureus* ATCC43300 (MRSA) (He et al., 2021).

Additionally, *A. flavipes* has been reported to produce a new indene derivative, methyl 2-(4-hydroxybenzyl)-1,7-dihydroxy-6-(3-methylbut-2-enyl)-1H-indene1-carboxylate, which inhibits the growth of *Klebsiella pneumoniae* and *P. aeruginosa* (Akhter et al., 2019). Moreover, bioactive extracts from *Aspergillus allahabadii* were investigated, revealing the production of the compound allahabadolactones B, exhibiting antibacterial activity against *Bacillus cereus* (Sadorn et al., 2016). Previous research has documented the antimicrobial, antioxidant, antidiabetic activities, and potential for biocontrol of *A. allahabadii* by various researchers (Affokpon et al., 2010; Sadorn et al., 2016; Rajamanikyam et al., 2017).

*A. fumigatus* possesses the ability to produce compounds like pseurotin A, isdethiobis(methylthio)gliotoxin, gliotoxin, spirotryprostatins A, and spirotryprostatins G, all displaying robust antimicrobial activity against pathogenic bacteria *E. coli*, *S. aureus*, and *C. albicans* (Zhang et al., 2018). Additionally, *A. fumigatus* is known for producing fumiquinazoline-F and fumiquinazoline-D, both exhibiting antibacterial and antifungal activities against *B. subtilis*, *S. aureus*, and *C. albicans* (Shaaban et al., 2013).

*A. micronesiensis*, an endophytic bacterium, produces cytochalasin A, a compound with antimicrobial activity against *S. aureus*, methicillin-resistant *S. aureus*, and *C. albicans* (Wu et al., 2019). Cytochalasans, a structurally diverse group of secondary metabolites, feature a substituted isoindole scaffold fused with a macrocyclic ring, showing a broad range of biological activities (Skellam, 2017). *A. nidulans* produces compounds such as 9-octadecenoic acid, methyl ester, methyl stearate, 9,12-octadecadienoic acid, 2-hydroxy-1-(hydroxymethyl) ethyl ester, and 9,17-octadecadienal, all inhibiting the growth of pathogenic bacteria including *S. aureus* ATCC 6538,*B. cereus* ATCC 10,987, *B. subtilis* ATCC 6633, *E. coli* ATCC 8739, *Salmonella typhimurium* ATCC14028, *Klebsiella pneumonia* ATCC 13,883, *P. aeruginosa* ATCC 9072, and *C. albicans* ATCC1023 bacteria (Sharaf et al., 2021). *A. niger* was further claimed to produce methylsulochrin compound. It is a diphenyl ether derivative that demonstrated antibacterial activity toward pathogenic bacteria, such as *S. aureus*, *Enterobacter cloacae*, and *Enterobacter aerogenes* (Mawabo et al., 2019).

**Fig. 3:**
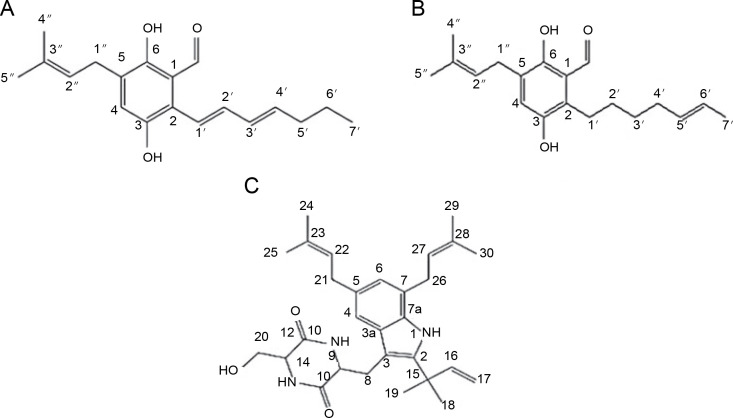
Structures of (A) dihydroauroglaucin, (B) isotetrahydroauroglaucin, and (C) cristatumin B (Elkady et al., 2022)

Elkady et al. (2022) successfully extracted endophytic metabolites from *A. niger* using ethyl acetate, identifying dihydroauroglaucin, isotetrahydroauroglaucin, and cristatumin B compounds (Fig. 3). Antibacterial tests revealed that dihydroauroglaucin exhibited broad antibacterial activity against carbapenem-resistant (CR) *K. pneumoniae*, methicillin-resistant *S. aureus* (MRSA), MDR *P. aeruginosa*, and vancomycin-resistant (VR) *Enterococcus faecalis*. Isotetrahydroauroglaucin was more effective against Gram-positive bacteria (MRSA and VR *E. faecalis*) and moderately active against Gram-negative bacteria (CR *K. pneumoniae* and MDR *P. aeruginosa* ). Additionally, cristatumin B displayed antibacterial activity across a wide spectrum targeting CR *K. pneumoniae* and MDR *P. aeruginosa* (Elkady et al., 2022).

**Fig. 4:**
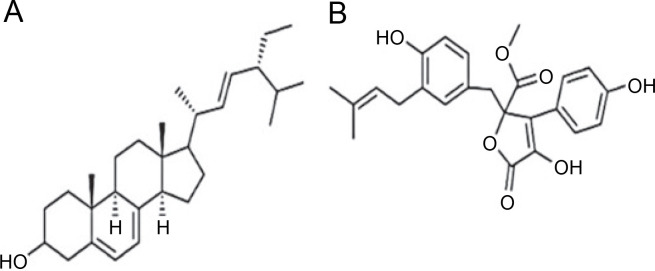
Structures of (A) (22E,24R) Stigmasta-5,7,22-trien-3β-ol (Elkhayat et al., 2016), (B) butyrolactone I compounds (da Silva et al., 2017)

*A. terreus* has been reported to produce (22E,24R) Stigmasta-5,7,22-trien-3β-ol (Fig. 4A), exhibiting antimicrobial activities against *S. aureus* and *C. neoformans* with IC_50_ values of 28.54 and 4.38 mg/ml, respectively (Elkhayat et al., 2016). (22E,24R)Stigmasta-5,7,22-trien-3b-ol was also effective against methicillin-resistant *S. aureus* (MRSA) with an IC_50_ of 0.96 mg/ml. Additionally, *A. terreus* produces aspernolides F (80), displaying antibacterial activity toward MRSA with an IC_50_ value of 6.39 mg/ml, and antifungal activity toward *C. neoformans* with an IC_50_ value of 5.19 mg/ml (Ibrahim et al., 2015; Elkhayat et al., 2016). da Silva et al. (2017) successfully extracted butyrolactone I from *A. terreus* using ethyl acetate, revealing its ability to inhibit *E. coli* (ATCC 25922) at a concentration of 117.6 μM (ATCC 25922).

It has been reported that *A. tubingensis* produced rubrofusarin B, 6-isovaleryl-4-methoxy-pyran-2-one, asperpyrones A (102), and campyrone A (103) compounds, inhibiting the growth of *E. coli*, *P. aeruginosa*, *S. lactis*, and *S. aureus* bacteria (Ma et al., 2015). Previous research by Yang et al. (2019) found that *A. tubingensis* produced the compound 3-(5-oxo-2,5-dihydrofuran-3-yl) propanoic acid, which exhibited antibacterial activity against *S. lactis*. Additionally, *A. tamarii* was reported to produce the compound disulfida cyclo-(Leu-Val-Ile-Cys-Cys) (1), known as malformin E, demonstrating significant antimicrobial activity against *B. subtilis*, *S. aureus*, *P. aeruginosa, E. coli, P. chrysogenum, C. albicans*, and *F. solani* (Ma et al., 2016).

### Penicillium genus

According to reports, endophytic *Penicillii* demonstrated the ability to colonize ecological niches and protect its host plant against various pressures by displaying a wide range of biological activities, applicable in agricultural, biotechnological, and pharmaceutical contexts (Toghueo and Boyom, 2020). The *Penicillium* genus, one of the largest fungal groupings, comprises over 200 identified species (Pitt et al., 2000). Metabolites produced by endophytic *Penicillii* play a crucial role in protecting host plants from pathogenic invasions, particularly through bioactive secondary metabolites with antimicrobial compounds. Yang et al. (2017) reported that *Penicillium* sp. MZKI P-265 produces helvolic acid, which exhibits inhibitory effects against *S. aureus* and *P. aeruginosa*.

The purified fermentation broth of *Penicillium* sp. HL4-159-41B contains the palitantin compound, showing promising activity in inhibiting the growth of *Mycobacterium tuberculosis* H37Ra (Li et al., 2015). Another research conducted by Jouda et al. (2016) revealed that penialidin A–C, citromycetin, p-hydroxyphenylglyoxalaldoxime, and brefeldin A have been successfully extracted from *Penicillium* sp. These compounds demonstrated the ability to inhibit the growth of *Vibrio cholerae* and *Shigella flexneri*, with penialidin C exhibiting the best antibacterial activity against *Mycobacterium smegmatis*. In addition, *P. cataractum*, another endophytic bacterium, was reported to produce penicimenolidyu A, penicimenolidyu B, and rasfonin compounds, all showing significant activity in inhibiting the growth of *S. aureus* (Wu et al., 2018).

Four compounds produced by *P. ochrochloron* (6-(2 R-hydroxy-3 E,5 E-diene-1 -heptyl)-4-hydroxy-3-methyl-2Hpyran-2-one, 6-(2 S-hydroxy-5 E-ene-1 -heptyl)-4-hydroxy-3-methyl-2H-pyran-2-one, 6-(2 S-hydroxy-1 -heptyl)-4-hydroxy-3-methyl-2H-pyran-2-one, and trichodermic acid) were tested for their activity against pathogenic bacteria. The results indicated antibacterial activity against *B. subtilis*, *Micrococcus luteus*, *S. aureus*, *Bacillus megaterium*, *Salmonella enterica*, *Proteus vulgaris*, *Salmonella typhi*, *P. aeruginosa*, *E. coli*, and *Enterobacter aerogenes* (Zhao et al., 2019). Additionally, another research report claimed that *P. ochrochloron* produced 4-O-desmethyl-aigialomycin B, penochrochlactones C, and penochrochlactones D, all of which demonstrated the ability to inhibit *S. aureus*, *B. subtilis*, *E. coli*, and *P. aeruginosa* (Song et al., 2021).

Moreover, *P. janthinellum* was reported to produce brasiliamide J-a & brasiliamide J-b, peniciolidone, and dehydroaustinol. Brasiliamide J-a & J-b compounds can inhibit the growth of *S. aureus*, *B. subtilis*, *P. aeruginosa*, *K. pneumonia*, and *E. coli*, while peniciolidone and dehydroaustinol were found to inhibit *S. aureus*, *B. subtilis*, and *E. coli* (Xie et al., 2018). *P. citrinum* was able to produce citrinin and emodin; citrinin exhibited antifungal activity against the plant pathogenic fungus *Alternaria citri*, while emodin had antifungal activity against the pathogenic fungus *Bipolaris maydis* (Luo et al., 2019).

*Penicillium setosum* was reported to synthesize kaempferol, patulin, leucodelphinidin, quercetin, and dihydroquercetin compounds, all exhibiting antibacterial activity against *E. coli* and *S. aureus* (George et al., 2019). *Penicillium vulpinum* was found to contain (−)-3-carboxypropyl-7-hydroxyphthalide and (−)-3-carboxypropyl-7-hydroxyphthalide methyl ester compounds. Specifically, (−)-3-carboxypropyl-7-hydroxyphthalide inhibited the growth of *Bacillus subtilis*, *Shigella dysenteriae*, and *Enterobacter aerogenes* with an MIC value of 12.5–25 μg/ml Meanwhile, (−)-3-carboxypropyl-7-hydroxyphthalide methyl ester displayed antibacterial activity against *E. aerogenes* with a MIC value of 12.5 μg/ml (Qin et al., 2019). Qin et al. (2020) also reported the production of 10-demethylated andrastone A, 15-deacetylcitreohybridone E, citreohybridonol, andrastins A, and andrastins B by *P. vulpinum*. All these compounds displayed inhibitory activity against *B. megaterium*. Notably, citreohybridonol exhibited strong inhibitory activity against *B. paratyphosus* and moderate inhibitory activity against *E. coli* and *S. aureus*.

**Fig. 5:**
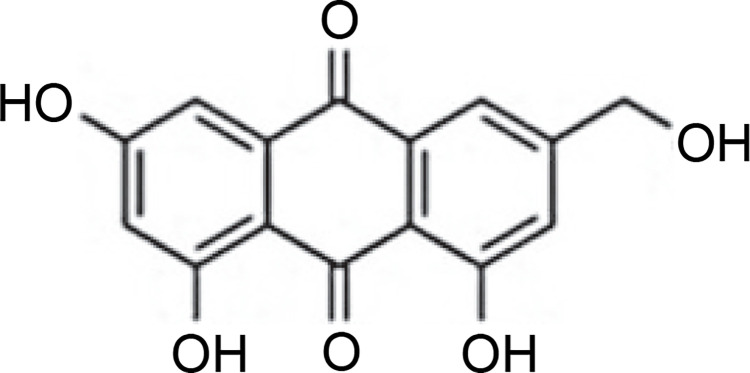
Structure of polyhydroxyanthraquinone compound (Graf et al., 2020)

*Penicillium vinaceum* produced (−)-(1R,4R)-1,4-(2,3)indolmethane-1-methyl-2,4-dihydro-1H-pyrazino-[2,1-b]-quinazoline-3,6-dione, an alkaloid quinazoline compound inhibiting the growth of *C. albicans* ATCC 76615 and *C. neoformans* ATCC 32609 (Zheng et al., 2012). *Penicillium brefeldianum* produced p-hydroxybenzaldehyde, a compound also found in *Syzygium zeylanicum* root bark that exhibited inhibitory activity against *S. typhi*, *E. coli*, and *B. subtilis* (Syarifah et al., 2021). *Penicillium restrictum* is known to produce polyhydroxyanthraquinone (Fig. 5), demonstrating inhibitory activity against MRSA (Graf et al., 2020). Furthermore, *Penicillium sumatrense* secreted citridone E and (–)-dehydrocurvularin compounds with antibacterial activity against *S. aureus*, *P. aeruginosa*, *Clostridium perfringens*, and *E. coli* (Xu et al., 2019).

**Fig. 6:**
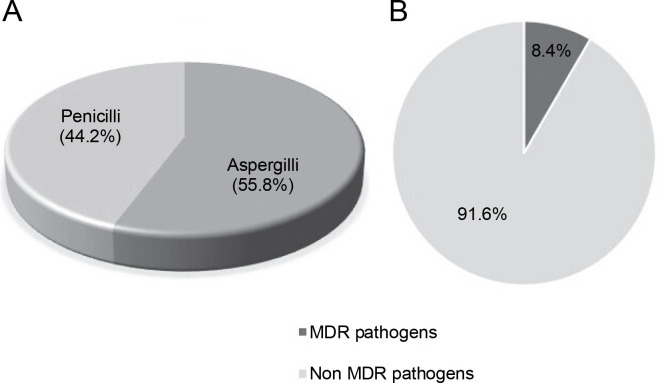
A) proportion of studies (*N* = 50) reporting *Aspergillii* and *Penicillii* from medicinal plants, B) proportion of studies (*N* = 50) reporting *Aspergillii* together with *Penicillii* targeting antibiotics-resistant and nonresistant microbes

Out of a total of 95 species of both *Aspergillii* and *Penicillii* known for their antimicrobial and multidrugresistant pathogen inhibitory activity, 55.8% were identified as *Aspergillus* and 44.2% as *Penicillium* (Fig. 6A). Further analysis of several articles revealed that endophytic fungi of *Aspergillus* and *Penicillium* inhibit multidrug resistant pathogens by only 8.4%, while the remaining 91.6% target nonMDR microorganisms (Fig. 6B).

Analysis of multiple articles indicates that certain species of *Aspergillus* and *Penicillium* possess antimicrobial activity against MDR pathogens. Consequently, it is imperative to continue exploring endophytic fungi to identify and assess their potential to produce bioactive compounds capable of eradicating pathogenic microorganisms, including MDR pathogens, in nature.

### Host plants

Endophytic fungi establish a mutually beneficial relationship with their host plants. They receive food and protection from the host plant, while the plant benefits from increased resistance to both abiotic and biotic stresses (Saikkonen et al., 2015; Mamangkey et al., 2022b).

Due to this symbiotic association, endophytic bacteria residing alongside plant tissues generate bioactive compounds akin to those synthesized by the plants themselves (Mamangkey et al., 2019; Munir et al., 2019). Medicinal plants are widely valued for their potential in disease prevention, owing to their renowned abundance of natural constituents (Yirga et al., 2011; Pan et al., 2013). Ultimately, these medicinal plants serve as natural habitats for the colonization of endophytic fungi, which, in turn, produce multifunctional compounds.

Endophytic bacteria and fungi are microorganisms that can be isolated from various parts of medicinal plants, including roots, stems, leaves, fruits, seeds, rhizomes, and tree barks (Mamangkey et al., 2019; Mamangkey et al., 2020; Mamangkey et al., 2022a). While both categories of endophytes, namely bacteria and fungi, are capable of producing bioactive compounds, fungi are more commonly isolated and have exhibited a higher propensity for generating a broader array of secondary metabolites. This remarkable diversity significantly enhanced the likelihood of uncovering novel antibacterial agents (Radić and Strukelj, 2012). Endophytic fungi that produce bioactive chemicals have a wide range of inherent qualities, including anti-inflammatory, antimicrobial, anticancer, antidiabetic, and antibiotic (Ruma et al., 2013; Mamangkey et al., 2022a, 2022b, 2023). The tabulation of information and reports on several documented *Aspergillii* and *Penicillii* originating from medicinal plants can be seen in Table 1.

**Table 1:** Symbiotic *Aspergilli* and *Penicilli* species in medicinal plants

Endophyte fungi	Host species	Reference
*Aspergillus* sp. TJ23	*Hypericum perforatum*	Qiao et al., 2018
*Aspergillus* sp. DTE1	*Medinilla speciosa*	Amelia et al., 2021
*Aspergillus* sp. TR_L1	*Tabebuia rosea*	Elkady et al., 2022
*A. ochraceus*	*Bauhinia forficate*	Bezerra et al., 2015
*A. neobridgeri*	*Pistacia lentiscus*	Sadrati et al., 2020
*A. niger* (OL519514)	*Opuntia ficus-indica*	Elkady et al., 2022
*A. versicolor*	*Eichhornia crassipes* *Pulicaria crispa*	Ebada and Ebrahim, 2020; Ibrahim and Asfour (2018)
*A. flavipes*	*Suaeda glauca*	Akhter et al., 2019
*A. flavus*	*Garcinia multiflora*	He et al., 2021
*A. allahabadii*	*Cinnamomum subavenium*	Sadorn et al., 2016
*A. flavus*	*Corchorus olitorius* *Mentha piperetta*	Chowdhury et al., 2018; Khattak et al. 2021
*A. fumigatus*	*Edgeworthia chrysantha* *Ipomoea batatas*	Zhang et al., 2018; Shaaban et al., 2013
*A. micronesiensis*	*Phyllanthus glaucus*	Wu et al., 2019
*A. nidulans*	*Ocimum basilicum*	Sharaf et al., 2021
*A. tamarii*	*Ficus carica*	Ma et al., 2016
*A. niger*	*Acanthus montanus* *Opuntia ficus-indica*	Mawabo et al., 2019; Elkady et al., 2022
*A. terreus*	*Carthamus lanatus* *Hyptis suaveolens*	Ibrahim et al., 2015; Elkhayat et al., 2016; da Silva et al. 2017
*A. tubingensis*	*Lycium ruthenicum* *Decaisnea insignis*	Ma et al., 2015; Yang et al., 2019
*A. tamarii*	*Ficus carica*	Ma et al., 2016
*Penicillium* sp.	*Aralia nudicaulis* *Garcinia nobilis* *Curcuma longa* *Melia azedarach* L. *Murraya paniculata* (L.) Jack *Schinus terebinthifolius* *Pinellia ternate*	Li et al., 2015; Jouda et al., 2016; Singh et al., 2014; Pastre et al., 2007; Tonial et al., 2016; Yang et al. 2017
*P. ochrochloron*	*Taxus media* *Kadsura angustifolia*	Zhao et al., 2019; Song et al., 2021
*P. janthinellum*	*Panax notoginseng*	Xie et al., 2018
*P. setosum*	*Withania somnifera*	George et al., 2019
*P. vulpinum*	*Sophorae tonkinensis*	Qin et al., 2019; Qin et al., 2020
*P. vinaceum*	*Crocus sativus*	Zheng et al., 2012
*P. brefeldianum*	*Syzygium zeylanicum*	Syarifah et al., 2021
*P. restrictum*	*Silybum marianum* (L) Gaertn.	Graf et al., 2020
*P. sumatrense*	*Garcinia multiflora*	Xu et al., 2019
*P. citrinum*	*Stephania kwangsiensis*	Luo et al., 2019
*P. cataractum*	*Ginkgo biloba*	Wu et al., 2018
*P. ochrochloronthe*	*Taxus media*	Zhao et al., 2019
*P. roqueforti*	*Solanum surattense*	Ikram et al., 2019
*P. minioluteum*	*Orthosiphon stamineus* Benth	Tong et al., 2014; Rozman et al., 2017
*P. amestolkiae*	*Orthosiphon stamineus* Benth	
*P. purpurogenum*	*Swietenia macrophylla*	Yenn et al., 2017
*P. setosum*	*Withania somnifera*	George et al., 2019

**Fig. 7:**
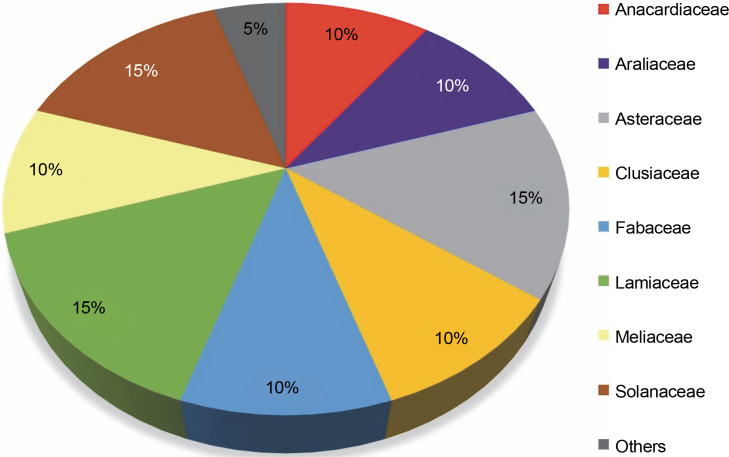
Proportion of plant species within families (*N* = 20) harboring endophytic *Aspergillii* and *Penicillii*

This review highlights 44 medicinal plant species that act as natural hosts for endophytic fungi, particularly within the *Aspergillus* and *Penicillium* genera. These plants span across 20 different families, as illustrated in Figure 7. Among these medicinal plant species, 15% prominent families have surfaced: *Asteraceae*, *Lamiaceae*, and *Solanaceae*, with each family comprising three species. Additionally, families such as *Anacardiaceae*, *Araliaceae*, *Clusiaceae*, *Fabaceae*, and *Meliaceae* each have 10% species, while 25 other medicinal plant families have 5% species. The prevalence of *Aspergillus* and *Penicillium* endophytes in these 44 medicinal plant species, especially within the *Asteraceae*, *Lamiaceae*, and *Solanaceae* families, suggests that these plants contain a diverse range of bioactive compounds effective against pathogenic fungi and bacteria, including MDR pathogens. These plants likely possess a well-organized system of chemical defense mechanisms, enabling them to protect themselves from diseases and environmental stress. Simultaneously, they offer a potential source of novel drugs for human use.

Research has indicated the presence of endophytic microbiota in various plant groups, from thallophytes to spermatophytes, and across diverse habitats from hydrophytes to xerophytes (Verma et al., 2017a, 2017b, 2017c). Caruso et al. (2020) highlighted the presence of various medicinal plant species within the *Asteraceae* family. Numerous studies have documented the pharmacological activity and chemical constituents of *Asteraceae* plants, revealing the presence of polyphenols, sesquiterpenes, organic acids, and fatty acids. These compounds have been associated with the effective treatment of various health conditions, including cardiovascular diseases, cancer, microbial and viral infections, inflammation, and other ailments (Morales et al., 2014).

The *Lamiaceae* (Labiatae) family, known as “lumbase nilcols” in Asian countries, stands out as a rich source of essential oils extensively utilized in the food, pharmaceutical, and cosmetic industries. These plants are characterized by secondary metabolites with antibacterial, antifungal, antioxidant, anti-inflammatory, and antiviral properties (Mesquita et al., 2019; Mamangkey et al., 2023). Typically, endophytic bacteria produce these secondary metabolites, utilizing plant cells as a source of nutrition. The *Solanaceae* family, comprising over 2000 species across 90 genera, has substantial commercial value in both the food and medicinal industries, as reported by Petkova et al. (2021). Previous research on other *Solanaceae* members has revealed systematic colonization by endophytic fungi within these plants (Tefera and Vidal, 2009; Jaber and Enkerli, 2016; Jaber and Araj, 2018). The ability of endophytic fungi to colonize *Solanaceae* plants suggests that specific growth requirements are adequately met within this family.

## Conclusion

*Aspergillus* and *Penicillium* endophytic fungi are recognized for their antimicrobial properties, particularly against multidrug-resistant pathogens. Among the medicinal plants under study, those belonging to the *Asteraceae*, *Lamiaceae*, and *Solanaceae* families have garnered significant attention due to their association with these endophytes. The rich diversity of bioactive compounds systematically synthesized within these plants may elucidate the potential of endophytic *Aspergillii* and *Penicillii* fungi in inhibiting and eradicating pathogenic microbes. As a result, these fungi have the potential to serve as sources for new antibiotics, offering potential solutions for controlling infections caused by MDR pathogens. The development of novel antibiotics from endophytic fungi associated with medicinal plants is an exciting prospect for the pharmaceutical industry.
